# Maternal and Neonatal Tetanus Elimination (MNTE) in The WHO African Region

**Published:** 2018-07-28

**Authors:** Eshetu Shibeshi Messeret, Balcha Masresha, Ahmadu Yakubu, Fussum Daniel, R Mihigo, Deo Nshimirimana, Joseph Okeibunor, Batholomew Akanmori

**Affiliations:** 1 Inter-country Support Team of East and Southern Africa, WHO African Region, Harare, Zimbabwe; 2 Immunization and Vaccines Development Programme, Family & Reproductive Health Cluster, WHO African Region, Brazzaville, Congo; 3 Immunization Vaccines and Biologicals Department, WHO Headquarters, Geneva, Switzerland; 4 WHO Country Office, Dakar, Senegal; 5 Polio Eradication Programme, WHO African Region, Brazzaville, Congo

**Keywords:** Elimination, Maternal And Neonatal Tetanus, WHO, African Region

## Abstract

Tetanus is a vaccine-preventable disease of significant public health importance especially in developing countries. The WHO strategy for the elimination of maternal and neonatal tetanus recommends the promotion of clean delivery practices, systematic immunization of pregnant women and those in the reproductive age (15-49 years) and surveillance for neonatal tetanus. Implementation of the recommended strategy with the support of WHO, UNICEF and other partners has led to significant decline in number of cases and deaths due to NT over the last decades. The coverage with the second or more dose of tetanus toxoid-containing vaccines (TT2+) a proxy for Protection at Birth (PAB) for the WHO African region has risen from 62% in 2000 to 77% by 2015 Reported cases of NT declined from 5175 in 2000 to 1289 in 2015.

The goal of eliminating maternal and neonatal tetanus by 2015 was missed, but some progress has been made. By the end of 2016, 37 out of 47 (79%) of the WHO AFR member states achieved elimination. The 10 member states remaining need additional support by all partners to achieve and maintain the goal of MNTE. Innovative ways of implementing the recommendations need to be urgently considered.

## Introduction

Tetanus is a non-communicable disease caused by a potent neurotoxin produced by *Clostridium tetani* bacteria whose resistant spores are present in soil and the environment. The causative organism is harboured by humans and animals that excrete the bacteria and spores. Once in a suitable anaerobic environment such as a contaminated wound, the bacteria multiply, releasing tetanus toxin, which is responsible for the symptoms and outcomes of the disease^1^,^2^. In the case of neonatal tetanus, the entry point for the bacteria is the umbilical stump. Some of the common traditional birth practices such as cutting the umbilical cord with unsterile instruments and the application of traditional substances (herbs, cow dung, rat faeces) onto the umbilical stump expose the newborn child to contamination with spores, thereby increasing the likelihood of infection and hence disease.

Despite decades of efforts to control and eliminate the disease, it still remains a major public health problem especially in remote areas with poor health care delivery and among the poor and illiterate populations. For instance a study in Benin City, Nigeria, identified lack of awareness of antenatal care services among the target population, under-utilisation of antenatal service, non-immunisation with tetanus toxoid vaccines, negative cultural beliefs, primordial cord care, lack of economic and decision making empowerment of the target population and lack of government commitment towards elimination of neonatal tetanus, as contributing towards the burden of neonatal tetanus in the area^3^. As a result, maternal and neonatal tetanus (MNT) is widely acknowledged as one of the key indicators of inequity in the provision of, health-care services, as it is extremely common in remote areas with poor health care delivery and among the poor and illiterate sections of the community^4^. Prevention of MNT requires minimizing contamination of wounds with bacterial spores through clean delivery as well as vaccination of women of reproductive age to ensure that they have high enough antibody titres against the toxin to pass to the foetus during pregnancy and prevent the disease in newborn babies. These measures together with public health messages have, over the years, significantly reduced the burden of neonatal tetanus globally.

In the late 1980s, the World Health Organization (WHO) estimated that about 787,000 newborn babies died from tetanus within the first 28 days of their lives. The most recent estimate for 2015 has shown a decline in the estimated NT deaths to 34,000, which is a 96% decline^12^. Even though the burden of disease has dropped significantly, MNT remains a significant public health problem especially in developing countries. Intense advocacy and sensitization of global leaders at several fora in the 1970s to the 1980s led to the World Health Assembly’s call for the elimination of neonatal tetanus (NT) by 1995^5^,^6^. By the year 1999, 104 of 161 developing countries had achieved elimination of NT. In the same year, the global maternal and neonatal tetanus elimination (MNTE) initiative was launched by WHO, United Nations Children’s Fund (UNICEF) and the United Nations Population Fund (UNFPA). with a commitment to jointly spearhead the efforts towards elimination of MNT by 2015. In line with the global targets, the WHO African Region (AFR) developed a strategy to accelerate the achievement of maternal and neonatal elimination (MNTE) and supported member states to implement national plans to achieve and validate elimination through neonatal mortality surveys.

The WHO defines neonatal tetanus elimination as the occurrence at the district level of less than 1 case of NT per 1000 live births annually. Maternal and neonatal tetanus is thus considered eliminated when neonatal tetanus cases are below the defined threshold.

The WHO strategy recommends the promotion of clean delivery practices, aimed at minimizing bacterial contaimination. It also reccomends systematic immunization of pregnant women and those in the reproductive age (15-49 years) with a tetanus toxoid-containing vaccine. Additonally, it reccomends theprovision of at least 3 doses of tetanus toxoid to women of reproductive age that reside in areas classified as being at high risk through supplemental immunization activities’ (SIAs). The vaccination will ensure that women have high enough antibodies to pass to their unborn babies during pregnancy to prevent the disease in the first few weeks after delivery when the risk is highest, in the event of bacterial contamination. Finally, case-based surveillance is used to identify NT cases and deaths as well as for risk assessment of populations^7^.

Progress in elimination has been slow and the AFR region missed its target by end-2015, due to slow implementation of the recommendations of the elimination strategy. As at end of 2016, ten of the remaining 18 countries where NT remains a public health problem (Angola, Chad, Central African Republic, DRC, Ethiopia, Guinea, Kenya, Mali, Nigeria and South Sudan) are in the African region. The manuscript summarizes the progress made in MNTE, highlights the challenges and suggets ways to acelerate the elimintion of the disease.

## Methods

The review of the progress made in the elimination of maternal and neonatal tetanus in the African Region of the World Health Organization is based on the evaluation of the implementation of the WHO strategy for MNTE by Member States.

### Key components of the strategy

The key components of the strategy are the promotion of clean delivery practices, systematic immunization of pregnant women and those in the reproductive age (15-49 years) with a tetanus toxoid-containing vaccine in routine immunization, or provision of at least 3 doses of tetanus toxoid through SIAs to women of reproductive age that reside in areas classified as being at high risk for MNT and the use of case-based surveillance to identify NT cases and deaths.

The coverage with the second dose of tetanus toxoid (TT2+) in pregnant women serves as a proxy for Protection at Birth (PAB). WHO and UNICEF obtain data from countries, which together with surveys is used to establish an estimate of the coverage with TT2^9^. To improve immunity among women of reproductive age residing in areas classified as high risk for tetanus, SIAs are organized with the support of WHO, UNICEF and other partners. These campaigns are often monitored to measure their quality and to address shortcomings. The joint WHO- UNICEF reporting form captures the cases of NT. This reflects protection and a measure of the progress in implementation.

The results presented reflect data obtained on the implementation of these activities by member States as reported in the WHO/UNICEF Joint Reporting Form.

## Results

**Figure 1:**
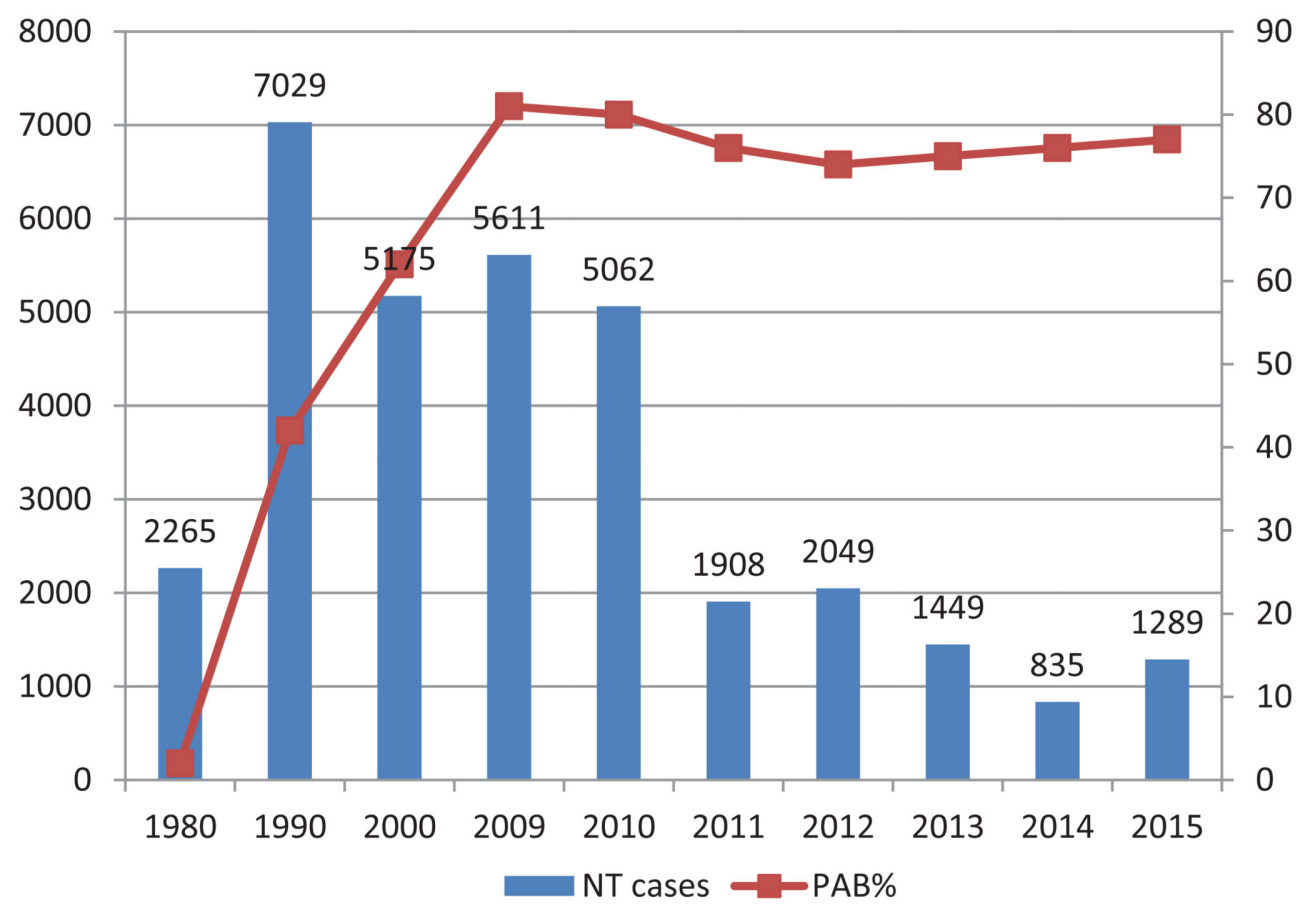
AFR Reported NT cases and % estimated coverage of protection at birth, 1980-2015 (Source WHO Regional summary 2015)

Figure 1 shows the number of officially reported NT cases and the proportion of women with PAB for the African Region, for the period 1990 - 2015. As per the WHO-UNICEF coverage estimate, coverage with the second dose of tetanus toxoid (TT2+), a proxy for Protection at Birth (PAB) was estimated at 62% in 2000 as compared to 77% by 2015 As a result of the progress made in routine immunisation and through the SIAs, a total of 1289 NT cases were reported in 2015 as compared to 5175 cases reported in 2000 through the joint WHO-UNICEF reporting form, indicating a decline of cases^10^.

The high-risk approach targets areas prioritized as at risk of maternal and neonatal tetanus. These countries have been conducting at least three rounds of SIAs. Between 2000 and 2015 a total of 78,985,175 women of reproductive age received at least two doses of Tetanus Toxoid in 31 countries of the WHO African Region.

The level of skilled delivery remains low and needs to be increased to sustain the gains achieved towards elimination. Skilled delivery rates rose from 36% in 2000 to 49% in 2015 in East and Southern Africa regions while it moved from 36% to 54% within the same period in West and Central African regions of UNICEF indicating that skilled delivery rate is still low in countries of the WHO African region^13^.

## Discussion

The member states of the AFR region have made remarkable progress towards achievement of the goal of MNTE. Of the 41 countries that attained MNTE from 2000 to 2016 out of 59 priority countries globally, 28 (68%) are in the AFR region. This is in addition to the nine countries that were already classified as having achieved elimination in 1999. These countries are also being supported to sustain their efforts so as to maintain their MNT elimination status. This support and guidance include a shift from the use of TT-only vaccine to Td vaccine given as a booster in schools and to pregnant women during antenatal care.

The remaining 10 member states that are yet to attain MNTE have their plans of action as part of the comprehensive multi-year plan for immunization and are at different levels of strategic implementation of their planned activities to achieve elimination.

Despite the apparent progress made in MNTE some challenges have been identified. The lack of awareness of antenatal services, under-utilisation of services and the sub-optimal integration of services with immunisation. These challenges lead to missed opportunities including non-immunisation with tetanus toxoid during antenatal care services. Other challenges are negative cultural beliefs, primordial cord care, lack of economic and decision making, empowerment of the target population and lack of government commitment towards elimination of neonatal tetanus, all affect the pace of elimination^3^.

Efforts are continuing through the scale up of the Reaching Every District approach in member states where most have adopted the 5-dose schedule in their immunization programmes to improve coverage with at least two protective doses of TT-containing vaccine. Based on the WHO strategic guidance countries have carried out surveillance and prioritized high-risk districts for NT cases. Using the surveillance and other core and surrogate data obtained the countries conduct at least three rounds of TT SIAs targeting women of reproductive age (15-49 years of age) in all districts classified as being at high risk for MNT. The aim of vaccination is to reach at least 80% of all women of reproductive age with three doses of the vaccine. Vaccination will ensure that the women have high enough antibodies against tetanus toxin to pass on to unborn children during pregnancy, which will protect against disease in the first few weeks of life when the risk is highest. Member states have implemented this strategy to ensure that targeted women in high-risk districts were reached. About 79 million women of reproductive age in the AFR region received at least two doses of TT from 2000 to 2015.

An important component of the MNTE strategy is surveillance for cases and deaths due to NT. The Integrated Disease Surveillance and Response (IDSR) is the main strategy that is being followed for disease notification, reporting, and action in the region. Efforts are ongoing to integrate NT surveillance into the active acute flaccid paralysis (AFP) surveillance for Polio using the vast infrastructure already in place. However, more cases are being documented through the IDSR than through the case-based surveillance for NT and cases are not followed by the appropriate response. Additionally, a significant number of the NT cases being reported through routine surveillance have been found not to be truly NT cases during programme reviews, pre-validation assessments or validation surveys, but more cases compatible with neonatal infections especially neonatal sepsis. More needs to be done in surveillance to obtain reproducible data which will inform programme implementation.

Although vaccination of pregnant women or women of reproductive age stands out as the most important intervention, regular antenatal check-ups, safe and clean deliveries also significantly contribute to the prevention of neonatal tetanus^11^. Thus, WHO is promoting clean delivery as an effective way to reduce maternal and neonatal infections, including tetanus. Improving maternal health has been given high priority by initiatives such as the Safe Motherhood Initiative. But with only 30%-50% of births attended by skilled health personnel in the least developed countries, there are still numerous challenges ahead. Distribution of clean delivery kits, community education, and training of skilled birth attendants (midwives, nurses, doctors) are some examples of how delivery practices are being improved.

The shortage of midwives, cultural preferences of the location of births, economic factors and attitude of health staff are, among others, some of the reasons for the low skilled attendants at birth rate in AFR region.

The manuscript has provided a brief update on the progress made in MNTE. However the paper is limited to only information obtained from the JRF, including coverage data which is not obtained from coverage surveys. The degree of accuracy of data is therefore limited. In addition, data may not have come from all parts of the countries including remote hard to reach or populations with poor access to health services, which are also the high risk areas for MNTE. *The goal of eliminating maternal and neonatal tetanus by 2015 has been achieved by 35 out of 47 (75%) of the WHO African Region member states. Two more countries attained MNTE in 2016, and* the remaining 10 member states need to be supported to use the high-risk approach, including increasing funding, to achieve the elimination goal while those that have achieved the goal need to sustain the gains through the implementation of appropriate strategies depending on their local context.

Rumours have circulated in some countries of the regions in the past, which have alleged that the vaccine against tetanus is meant to control birth. Attention should therefore also be paid to communicating factually and effectively about the immense value of vaccination of women of childbearing age in preventing disease and deaths from NT. Effective reporting about vaccine safety particularly among adolescents will minimize hesitancy which can cause reduction in coverage.

Additionally, surveillance for NT needs to improve substantially to include local response vaccination in the area that identified NT cases and community surveillance. This will help to bring the countries back on track to meeting the overall goal of MNT elimination.
